# Hypodermic Use of Morphia

**Published:** 1878-07

**Authors:** H. L. Harrington

**Affiliations:** Warren County, Illinois


### NOTES FROM PRIVATE PRACTICE.

*Hypodermic use of Morphia.*

Several articles have appeared in this Journal, relative to the use of hypodermic injections of morphia, in the main condemning it. The reports of cases by Doctor Wenger (see August No., 1877) seem to be quite indefinite, as neither the size of dose, nor a *complete* history of symptoms, were given. In the same article, Dr. Ingals reports a case, saying, " I dissolved one-fourth of a grain of morphia in pure water," etc., the patient almost immediately developing the most alarming symptoms. May not the syncope in this case have been due to other causes ? Many persons faint upon much less provocation. In Dr. Brown-Séquard's lecture, published in the May No. of this journal, page 452, he says : " The patient was to be bled ; the lancet had not gone in before the patient fainted away, the heart ceased to beat, and the limbs seemed to be dead. The patient was recalled to life with the greatest difficulty. Death would have occurred if the most energetic means had not been employed immediately." Is not this case almost exactly analagous to Dr. Ingals' ? In summing up the writer says : " The danger seems to arise from rapid absorption, or injection directly into the circulation," etc. Is it possible, that out of the vast number of injections used, in so few cases rapid absorption occurs ? I have never seen any marked difference in the *degree* of effect, which seemed to correspond with the rapidity of absorption. Again we find, " Nothing in the patient's history or general appearance can warn us of the danger ; " and, " The results of my inquiries seem to leave us no other choice than reasonable doses by the stomach, or the frequent repetition of very small doses hypodermically." *Do* the results of his inquiries warrant such positive assertions ? Do we know all that is to be learned about the subject ? The fact is patent enough, that negative evidence is not as valuable as positive, provided always that the latter *is* positive. The doubt implied in the last sentence is my excuse for relating my own experience.

During six months in 1874, that I was in the wards of Cook County Hospital daily with Dr. Steele, then resident physician, and during eighteen months of service as a member of the house staff of the same hospital, one year as resident physician and surgeon, I saw used, and administered myself, almost daily, sometimes many times daily, hypodermic injections of morphia, without a single fatal result, or any unpleasant symptom other than nausea, except in two cases.

A man aged about 40, the subject of serious lesion of both mitral and aortic valves, with dilatation and hypertrophy, and suffering from many of the severe symptoms usually attendant thereupon, was given several times, (injudiciously I now think) for the relief of very severe angina pectoris, injections of one-half grain of morphia. Upon one occasion, having instructed the nurse to watch him carefully, in about four hours' information came that the patient was as he thought, dying, I found him in a deep sleep, pulse 36 to 40, respirations 4 to 6 per minute: roused him without great difficulty and administered aromatic spirits of ammonia and whiskey, which were all that was required. The second case, an hysterical girl, suffering from facial neuralgia, was given three eighths of a grain, the solution entering a vein, as was evident from the fact that a drop of venous blood followed the withdrawal of the needle, and from the rapidity of its effect, the patient throwing up her hands and exclaiming, "oh, my head!" and acting in a delirious manner. Relief from pain and sleep followed much sooner than I ever saw it before or since, but no coma, syncope, or other serious results obtained. In private practice I have repeatedly used this method, always with relief of pain and no bad results, the dose varying from one quarter to three eighths of a grain, so large, simply because smaller amounts do not produce the desired effect. I have never seen the characteristic effects of the drug follow in a "few seconds" or "immediately," as some of the reports indicate, except in the last of the two cases, when a vein received the injection. Is it possible that absorption from the areolar tissue can take place and give rise to such formidable results in a second or two? Is it not more reasonable to suppose that some other condition gives rise to the symptoms? Again, we find the writer using these words: "The method is of little importance provided it is safe and agreeable to the patient."

Is this entirely true? Is it of *little* importance, whether the agony we are sometimes called upon to witness, is relieved in five or ten minutes or not for two or three hours, and perhaps not at all ? The well known depressing power of pain, and more, its power to *kill*, is a strong argument against this assertion.

May not these "representative physicians" have been a little careless in administering the remedy in question, either in the size of the dose used, or in not recognizing or attaching sufficient weight to some pathological condition, or idiosyncrasy, which should have deterred them from its use? One of them gave it when the pain was already relieved, just to make the patient sleep; another used ten drops of a solution in which the dose "probably equalled one and one-half grains, and may have been six and one-half grains." another uses an injection in a case of puerperal convulsions, in which probably there was already cerebral hyperæmia, (as Dr. Wenger had suggested bleeding), and the well known action of morphia would be to aggravate the trouble. *Were these men justified in so using the remedy.*

In order that physicians may not be *unnecessarily* deterred from using hypodermic injections, let all possible light be thrown upon the subject.

The ability to *stop pain* in a few minutes, is a boon worth years of discussion.

In conclusion, let me give the rules by which I am governed in its administration.

1. Never use hyperdermic injections, except for the relief of intense pain, or where the stomach will not retain the drug.

2. Have a solution, accurately prepared, so that the *exact* amount given, is in every instance known.

3. As morphine and pain are mutually antagonistic, and as it is well known that far larger doses are tolerated when pain is present, make the size of the dose proportionate to the severity of pain.

4. Do not leave the patient until sure that no unpleasant effects will follow.

H. L. HARRINGTON, M. D.

WARREN COUNTY, ILLINOIS.

